# Metabolic-Dysfunction-Associated Steatotic Liver Disease—Its Pathophysiology, Association with Atherosclerosis and Cardiovascular Disease, and Treatments

**DOI:** 10.3390/ijms242015473

**Published:** 2023-10-23

**Authors:** Hidekatsu Yanai, Hiroki Adachi, Mariko Hakoshima, Sakura Iida, Hisayuki Katsuyama

**Affiliations:** Department of Diabetes, Endocrinology and Metabolism, National Center for Global Health and Medicine, Kohnodai Hospital, 1-7-1 Kohnodai, Ichikawa 272-8516, Japan; dadachidm@hospk.ncgm.go.jp (H.A.); d-hakoshima@hospk.ncgm.go.jp (M.H.); d-20iida@hospk.ncgm.go.jp (S.I.); d-katsuyama@hospk.ncgm.go.jp (H.K.)

**Keywords:** cardiovascular disease, fatty acids, insulin resistance, metabolic-dysfunction-associated steatotic liver disease, pemafibrate, triglyceride

## Abstract

Metabolic-dysfunction-associated steatotic liver disease (MASLD) is a chronic liver disease that affects more than a quarter of the global population and whose prevalence is increasing worldwide due to the pandemic of obesity. Obesity, impaired glucose metabolism, high blood pressure and atherogenic dyslipidemia are risk factors for MASLD. Therefore, insulin resistance may be closely associated with the development and progression of MASLD. Hepatic entry of increased fatty acids released from adipose tissue, increase in fatty acid synthesis and reduced fatty acid oxidation in the liver and hepatic overproduction of triglyceride-rich lipoproteins may induce the development of MASLD. Since insulin resistance also induces atherosclerosis, the leading cause for death in MASLD patients is cardiovascular disease. Considering that the development of cardiovascular diseases determines the prognosis of MASLD patients, the therapeutic interventions for MASLD should reduce body weight and improve coronary risk factors, in addition to an improving in liver function. Lifestyle modifications, such as improved diet and increased exercise, and surgical interventions, such as bariatric surgery and intragastric balloons, have shown to improve MASLD by reducing body weight. Sodium glucose cotransporter 2 inhibitors (SGLT2i) and glucagon-like peptide-1 receptor agonists (GLP-1RAs) have been shown to improve coronary risk factors and to suppress the occurrence of cardiovascular diseases. Both SGLT2i and GLP-1 have been reported to improve liver enzymes, hepatic steatosis and fibrosis. We recently reported that the selective peroxisome proliferator-activated receptor-alpha (PPARα) modulator pemafibrate improved liver function. PPARα agonists have multiple anti-atherogenic properties. Here, we consider the pathophysiology of MASLD and the mechanisms of action of such drugs and whether such drugs and the combination therapy of such drugs could be the treatments for MASLD.

## 1. Introduction

The principal limitations of the terms nonalcoholic fatty liver disease (NAFLD) and nonalcoholic steatohepatitis (NASH) are the reliance on exclusionary confounder terms and the use of potentially stigmatizing language. The name chosen to replace NAFLD was metabolic-dysfunction-associated steatotic liver disease (MASLD) [1]. There was consensus to change the definition to include the presence of steatotic liver disease and at least one of five cardiometabolic risk factors, which include (1) increase in body mass index (BMI) or waist circumference (WC); (2) impaired glucose metabolism; (3) high blood pressure; (4) high triglyceride (TG) levels; (5) low high-density cholesterol (HDL-C) levels [1]. The epidemiology and demographic characteristics of MASLD vary worldwide, usually parallel to the prevalence of obesity; however, a substantial proportion of patients are lean [2]. Individuals with MASLD have a high frequency of metabolic comorbidities and could place a growing strain on health-care systems all over the world. Prevalence data from 245 articles involving 2,699,627 persons were used with a hierarchical Bayesian approach to forecast the prevalence of MASLD through to 2040 [3]. By 2040, over half the adult population is forecasted to have MASLD [3]. The pandemic of obesity and its cardiometabolic consequences contribute to an increased prevalence of MASLD [4]. Approximately 20–30% of MASLD patients develop metabolic-dysfunction-associated steatohepatitis (MASH), leading to liver cirrhosis and associated complications, including hepatocellular carcinoma [5]. The worldwide disease burden from liver fibrosis due to MASLD is expected to increase around two to three-fold within a decade.

Furthermore, MASLD patients have cardiometabolic risk factors and are predisposed to liver fibrosis as well as atherosclerotic cardiovascular disease (ASCVD). Yoneda, M. et al. retrospectively analyzed data for 2,452,949 people to estimate the relationship between CVD and MASLD [6]. The incidence rates of CVD were 1.01 (95%CI (confidence interval), 0.98 to 1.03) and 2.69 (95%CI, 2.55 to 2.83) per 1000 person-years in the non-MASLD and MASLD groups, respectively. The overall prevalence of hypertriglyceridemia and diabetes was 13.6 and 4.3%, respectively, in the non-MASLD group and 64.1 and 20.6%, respectively, in the MASLD group. The CVD risk increased with hypertriglyceridemia and diabetes.

Therefore, for the treatment of MASLD patients, it is necessary to select the therapeutic strategies that protect not only the liver but also the cardiovascular system. Therefore, we discuss the shared pathological mechanisms regarding the development of MASLD and atherosclerosis. We also considered the anti-atherosclerotic effects of treatments currently considered to be effective in treating MASLD. Such considerations may contribute to an improvement in prognosis for MASLD patients.

## 2. The Metabolic Abnormalities That Induce MASLD

The features of metabolic syndrome are not only highly prevalent in patients with MASLD but components of metabolic syndrome also increase the risk of developing MASLD [5]. The established conditions for developing MASLD include obesity, type 2 diabetes, hypertension and dyslipidemia such as high TG and low HDL-C levels [5].

Obesity is the most common and well-documented risk factor for MASLD. To investigate whether central obesity is associated with MASLD formation after adjusting for general obesity, a meta-analysis was performed [7]. In the meta-analysis, which used twenty eligible studies, the pooled odds ratio (OR) in WC and BMI were 2.34 (95%CI, 1.83 to 3.00) and 2.85 (95%CI, 1.60 to 5.08), respectively [7].

Although MASDL, MASH and MASH with advanced fibrosis are closely associated with type 2 diabetes, their global prevalence rates have not been well described. To estimate the prevalence of MASLD, MASH and advanced fibrosis among patients with type 2 diabetes, a meta-analysis using 80 studies from 20 countries was performed [8]. The global prevalence of MASLD among patients with type 2 diabetes was 55.5% (95%CI, 47.3 to 63.7) and the global prevalence of MASH among individuals with type 2 diabetes was 37.3% (95%CI, 24.7 to 50.0) [8].

An increasing body of evidence connects MASLD to hypertension. To estimate the nature and magnitude of the association between MASLD and hypertension, a meta-analysis using 11 studies was performed [9]. The presence of hypertension was significantly associated with an increased risk of incident MASLD (hazard ratio (HR), 1.63; 95%CI, 1.41 to 1.88) [9]. Pooled analysis showed that the presence of MASLD was significantly associated with an increased incidence of hypertension (HR, 1.55; 95%CI, 1.29 to 1.87) [9], indicating the existence of a bidirectional relationship between MASLD and hypertension that was independent of traditional cardiometabolic risk factors.

Dyslipidemia, which includes high serum TG levels and low serum HDL-C levels, is also common in patients with MASLD. The prevalence of MASLD in individuals with dyslipidemia attending lipid clinics has been estimated to be 50% [10].

Metabolic syndrome and its components such as obesity, impaired glucose metabolism, high blood pressure and dyslipidemia are all closely associated with insulin resistance. The pathogenesis of MASLD largely remains unknown. Sanyal, A.J. et al. demonstrated that peripheral insulin resistance was present in both MASLD and MASH patients by using a hyper-insulinemic euglycemic clamp [11]. Many investigations have shown that defects in the insulin signaling pathway, especially those associated with insulin receptor substrate-2 (IRS-2), are definitely implicated in the pathogenesis of insulin resistance [12]. Rats with MASLD developed insulin resistance, showing increased fasting blood glucose and insulin levels, increased weight of epididymal fat, obvious hepatic steatosis and inflammation and down-regulated IRS-2 mRNA and protein levels compared with normal controls [13]. Further, an insulin sensitizer, pioglitazone, underwent significant recovery, including up-regulated IRS-2 mRNA and protein levels [13]. Insulin resistance may greatly contribute to the development of MASLD.

**Figure 1:**
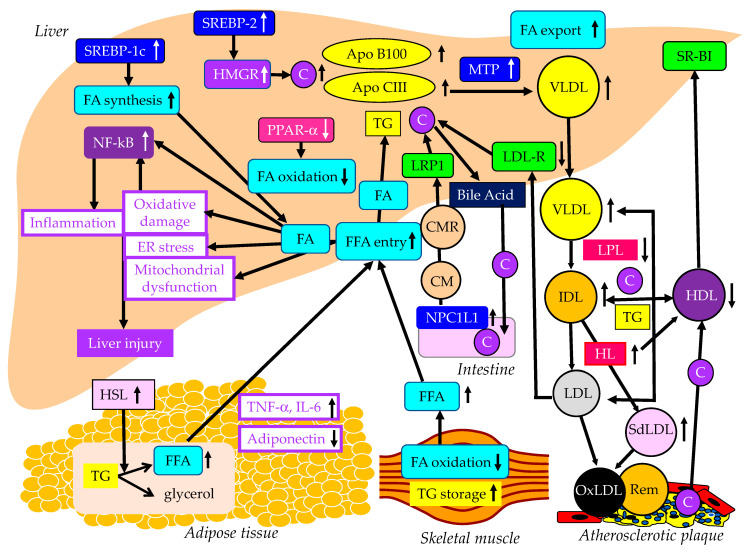
The abnormal lipid metabolism possibly induced by insulin resistance and its association with the development of MASLD. Black and white arrows pointing upward and downward indicate an increase or decrease in expression or activity, respectively. Solid black lines indicate the flow of substances and the effects of each metabolic event.

The effects of lipid metabolism abnormalities induced by insulin resistance on the development of MASLD are shown in Figure 1. Accumulated visceral adipose tissue produces more inflammatory cytokines, such as tumor necrosis factor alpha (TNF-a), interleukin-6 (IL-6) and IL-1b, and less adiponectin, which induces systemic insulin resistance [14]. The metabolism of free fatty acids (FFAs) is altered in insulin resistance [15]. The enzymes lipoprotein lipase (LPL) and hormone-sensitive lipase (HSL) are rate-limiting factors for TG and FA metabolism because LPL hydrolyzes extracellular TG in lipoproteins and HSL hydrolyzes intracellular TG in adipocytes [16].

Insulin resistance enhances the expression and activity of HSL in adipose tissue. HSL catalyzes the hydrolysis of TG into FFA [17]. Insulin resistance is closely associated with an excess TG storage within the skeletal muscle [16]. Insulin resistance reduces FA oxidation, leading to diminished use of FAs and storage of TG within the skeletal muscle. Serum FFAs increase due to increased release from the adipose tissue and decreased FA use in the skeletal muscle. An increased amount of FFA enters the liver, leading to overproduction of TG-rich lipoproteins such as very-low-density lipoprotein (VLDL). Insulin resistance is associated with reduced apo B100 degradation [18] and elevated hepatic apo CIII production [19], which increase VLDL because both apo B100 and apo CIII constitute VLDL. Insulin resistance increases the expression of microsomal TG transfer protein (MTP), a key enzyme involved in VLDL assembly [18]. In an insulin-resistant state, an increased FFA entry to liver, reduced degradation of apo B100 and enhanced expression of apo CIII and MTP may elevate hepatic production of VLDL. Insulin resistance also causes an increased expression of sterol regulatory element binding protein 1c (SREBP-1c), which increases FA synthesis [20]. Hepatic FA metabolism is regulated by a combination of FA uptake, FA export by VLDL secretion, de novo FA synthesis by SREBP-1c and FA utilization by β-oxidation. FA accumulation is one of the features of MASLD.

Two major physically distinct species of VLDL exist: larger TG-rich VLDL1 and smaller VLDL2 [21]. At normal TG concentrations, VLDL1 and VLDL2 circulate in approximately equal proportions. Hepatic TG accumulation and insulin resistance increase VLDL1 secretion [22,23]. MASH patients have been shown to have more pronounced postprandial intestinal and hepatic VLDL1 accumulation, LDL lipid peroxidation and reduced total antioxidant status [24]. Postprandial intestinal VLDL1 independently predicted oxidized LDL (OxLDL) and reduced total antioxidant status responses in MASH. Postprandial intestinal VLDL1 accumulation is associated with a pro-oxidant imbalance in MASH, and both correlate with the severity of liver disease. Otsuka Long-Evans Tokushima fatty rats showed overproduction of VLDL compared with control rats [25]. In the livers of these rats, the mRNA levels of TNF-α, IL-1b and IL-6 were increased and the mRNA, protein, and tyrosine phosphorylation levels of IRS-2 were decreased. Overproduction of VLDL in the liver is significantly associated with hepatic oxidative stress, inflammation and insulin resistance. However, it remains unclear whether VLDL itself has the property of enhancing such exacerbating factors of liver fibrosis or whether metabolic abnormalities that induce VLDL overproduction promote liver fibrosis. TG accumulation and VLDL overproduction are also features of MASLD.

MTP is predominantly expressed in hepatocytes and enterocytes and is required for the assembly and secretion of VLDL. A rare causal variant in the MTP gene that was associated with progressive MASLD, unrelated to metabolic syndrome, was identified [26]. Hepatocyte-like cells derived from a homozygote donor had significantly lower MTP activity and lower lipoprotein apo B secretion than wild-type cells. Cytoplasmic TG accumulation in hepatocyte-like cells triggered endoplasmic reticulum stress, secretion of pro-inflammatory mediators and production of reactive oxygen species (ROS). This MTP gene variant was associated with progressive MASLD. Increased expression of MTP can be beneficial for protection against MASLD. Cytoplasmic TG accumulation may induce MASLD.

FA oxidation primarily occurs in the mitochondria; however, FA oxidation commences in the peroxisomes and then is finally processed in the mitochondria [27]. In obesity, ω-oxidation by cytochrome P450 enzymes also contributes to FA oxidation. This pathway for FA oxidation generates large amounts of ROS [28]. The entry of FAs into mitochondria depends on carnitine palmitoyl-transferase 1 (CPT-1). One of the major regulators of CPT-1 is the peroxisome proliferator-activated receptor (PPAR)-α [29,30,31,32]. Activation of PPARα induces the transcription of genes related to FA oxidation [29,33,34]. Visceral adiposity and insulin resistance are negatively correlated with liver PPARα gene expression [34].

Overexpression of apo CIII, independent of a high-fat diet, produces MASLD-like features, including increased liver lipid content, decreased antioxidant capacity, increased expression of TNFα and IL-1β and decreased expression of adiponectin receptor [35]. A high-fat diet induced hepatic insulin resistance and marked increases in plasma TNFα (eight-fold) and IL-6 (60%) in apo-CIII-overexpressing mice [35]. Cell death and apoptosis were augmented in apo-CIII-overexpressing mice regardless of diet [35]. Fenofibrate treatment reversed several of the effects associated with diet and apo CIII expression but did not normalize inflammatory traits even when the liver lipid content was fully corrected [35]. An increase in apo CIII plays a major role in liver inflammation and cell death in MASLD. There are no reports on adverse effects for apo CIII deficiency in MASLD, and increased apo CIII may adversely affect MASLD.

An increase in FFAs leads to hepatic insulin resistance by interacting with insulin signaling [36,37]. The anti-lipolytic function of insulin is impaired in insulin resistance, which may facilitate hepatic TG synthesis. Saturated FAs generate lipotoxic intermediate products, such as diacylglycerols [38]. Lipotoxic intermediate products cause endoplasmic reticulum stress and ROS formation, which are major factors for the pathogenesis of MASH [39,40]. By binding to Toll-like receptor 4, saturated FAs induce the augmentation of mitochondrial dysfunction and activation of pro-inflammatory nuclear factor-kappa B (NF-κB) [39].

The studies using animal models, particularly those using molecular inhibition of TG synthesis [41], and the available small human lipidomic studies, have ruled out TG as the major lipotoxic mediator of MASH [42]. The focus now falls on other lipid species, particularly FFAs, diacylglycerol, toxic phospholipids (ceramides, sphingolipids) [42] and, most recently, cholesterol. Insulin resistance activates SREBP-2, which induces the expression of 3-hydroxy-3-methyl-glutaryl-CoA reductase (HMGR), the rate-limiting enzyme of cholesterol biosynthesis, resulting in increases in free cholesterol and cholesterol ester in the liver [43]. Such increased free cholesterol and cholesterol ester may induce inflammation and cell death [43].

## 3. The Association of MASLD with ASCVD

A retrospective analysis of 619 patients diagnosed with MASLD showed that CV events (38.3%), followed by non-liver malignancy (18.7%) and complications of liver cirrhosis (7.8%), were the three most common causes of death in MASLD patients [44], suggesting that CV events were the most crucial determinant of mortality in MASLD patients. A meta-analysis showed that MASLD was significantly associated with an increase in the development of CVD (odds ratio (OR), 2.05; 95% confidence interval (95%CI), 1.81 to 2.31; *p* < 0.0001) [45]. However, MASH has a higher liver-related (OR for MASH, 5.71; 95%CI, 2.31 to 14.13; OR for MASH with advanced fibrosis, 10.06; 95%CI, 4.35 to 23.25) but not cardiovascular mortality (OR, 0.91; 95%CI, 0.42 to 1.98). Therefore, patients with MASLD can be said to be a high-risk group for CVD as well as a high-risk group for developing MASH.

A multicenter large retrospective study showed that the BMIs of subjects with MASLD were significantly higher than in those without MASLD (*p* < 0.01) [46]. The prevalence of MASLD showed a linear increase corresponding to an increase in BMI (BMI < 23 kg/m^2^, 10.5%; BMI ≥ 23 kg/m^2^ and <25 kg/m^2^, 37.9%; BMI ≥ 25 kg/m^2^ and <28 kg/m^2^, 58.4%; BMI ≥ 28 kg/m^2^, 84.2%) [46]. In short, a 7.4–11.4% increase in the prevalence of MASLD per 1 kg/m^2^ of BMI was observed. The prevalence of MASLD showed a linear increase when serum TG and LDL-C levels were increased and a linear decrease when HDL-C levels were increased. The prevalence of MASLD was 22.8% in subjects with normal TG levels (<150 mg/dL) and 59.5% in subjects with hypertriglyceridemia (>150 mg/dL). The prevalence of MASLD was 27.3% in subjects with normal HDL-C levels (>40 mg/dL) and 61.7% in subjects with hypo-HDL-C (<40 mg/dL). The prevalence of MASLD was 26.4% in subjects with normal LDL-C (<140 mg/dL) and 38.5% in subjects with hyper-LDL-C (>140 mg/dL) [46].

The increased production of VLDL observed in MASLD is caused by insulin resistance as described above, and insulin resistance reduces the degradation of VLDL in the blood (Figure 1). Insulin resistance adversely affects enzymes such as LPL and hepatic lipase (HL), leading to conditions that are highly atherogenic, such as a decrease in HDL and increases in small-dense LDL (SdLDL) and remnant lipoproteins [47]. Insulin resistance reduces LPL activity [47]. LPL is the rate-limiting enzyme for the catabolism of TG-rich lipoproteins such as VLDL and intermediate-density lipoprotein (IDL) [48]. The formation of HDL is related to the catabolism of TG-rich lipoproteins by LPL [49]. Therefore, reduced LPL activity increases VLDL and IDL and reduces HDL. The activity of HL, the enzyme that facilitates the catabolism of HDL, is correlated with insulin resistance [50]. Low serum HDL-C levels may be partially due to an increased clearance by HL [50]. LDL size is inversely proportional to HL activity [51], and patients with high HL have more SdLDL as compared with subjects with low HL activity [52]. Increased HL activity due to insulin resistance may increase atherogenic lipoprotein and SdLDL. Remnant lipoproteins have undergone extensive intravascular remodeling. LPL, HL and cholesterol ester transfer protein (CETP) induce structural and atherogenic changes that distinguish remnant lipoproteins from non-remnant lipoproteins [21]. Via the LPL-mediated removal of TG and the CETP-mediated exchange of TG for cholesterol from LDL and HDL, remnant lipoproteins contain more cholesterol than nascent VLDL [53].

HDL plays a role in reverse cholesterol transport from atherosclerotic plaques, which is an anti-atherogenic effect [54]. Therefore, reduced HDL induces an atherogenic status. Since SdLDL is not recognized by the LDL receptor, SdLDL stays in blood for a longer period [54]. SdLDL is likely to be adhesive to the endothelium and migrate into the subendothelial space. SdLDL is easily oxidized because of a lack of antioxidative capacity [54]. LDL and SdLDL are taken up by macrophages via a scavenger receptor after oxidative modification [54]. Remnant lipoproteins are taken up by macrophages without modification such as oxidation, which is a highly atherogenic property.

Insulin modulates LDL receptor expression and activity. The inactivity of insulin represses LDL receptor transcription [55], which can explain the increase in LDL-C in the insulin-resistant state. Niemann-Pick C1-like 1 (NPC1L1) plays a pivotal role in intestinal cholesterol absorption. The expression of NPC1L1 was investigated in non-diabetic rats and diabetic cholesterol-fed rats [56]. There was a positive correlation between intestinal NPC1L1 mRNA and chylomicron (CM) cholesterol. LDL receptor-related protein 1 (LRP1) is an endocytic and signaling receptor expressed in several tissues that plays a crucial role in clearance of CM remnants from circulation [57]. Furthermore, LDL and other cholesterol-rich, apo B-containing lipoproteins, once they become retained and modified within the arterial wall, cause atherosclerosis [58].

## 4. The Therapeutic Approaches for MASLD—Lifestyle Modification and Surgical Interventions

Weight reduction and an improvement in atherogenic lipoproteins are important to improve the prognosis of MASLD patients. Therefore, lifestyle modification, such as improved diet and increased exercise, and surgical interventions to reduce body weight can be the promising therapeutic strategies for MASLD.

### 4.1. Lifestyle Modification

#### 4.1.1. Diet

Weight loss by lifestyle modification is the cornerstone therapy of MASLD. Low carbohydrate diets have showed favorable effects for body weight as well as hepatic fat content in several reports. In a meta-analysis, there was no significant difference between a low carbohydrate diet group and a low fat diet group in the improvement of hepatic fat content and liver enzymes in MASLD [59]. In a meta-analysis of eight randomized clinical trials (RCTs), the Mediterranean and hypocaloric dietary interventions favoring unsaturated FAs resulted in improvements in intrahepatic lipid content and liver enzymes in patients with MASLD [60]. Another meta-analysis showed that calorie-restricted interventions had favorable effects on alanine aminotransferase (ALT) (*p* < 0.001), hepatic steatosis (*p* < 0.001) and liver stiffness (*p* = 0.009) [61]. The Mediterranean diet reduced ALT (*p* = 0.02), the fatty liver index (*p* < 0.001) and liver stiffness (*p* = 0.05). There was a dose–response relationship between the degree of calorie restriction and the beneficial effects on liver function and weight loss.

Intermittent fasting, which includes alternate-day fasting, and other forms of periodic caloric restriction have already received attention from animal research scientists [62,63]. It has been shown that fasting may benefit weight management and improve cardiovascular and metabolic risks [64]. In the meta-analysis, there were significant differences in body weight, BMI and ALT and aspartate aminotransferase (AST) levels between the control and intermittent fasting groups [65]. In another meta-analysis, body weight, BMI and waist-to-hip ratio were significantly improved following the intermittent fasting intervention (*p* < 0.05) [66]. Adults with MASLD showed an improvement in serum ALT and AST levels, hepatic steatosis and hepatic stiffness measured by vibration-controlled transient elastography after an intermittent fasting intervention (*p* < 0.05) [66].

#### 4.1.2. Exercise

Increased physical activity, independently of diet change, was associated with a significant reduction in intrahepatic lipid content and with reductions in ALT and AST levels [67]. Individuals with increasing BMIs are more likely to benefit from the intervention. Compared with standard care, exercise improved serum ALT and AST levels and the amount of intrahepatic fat [68]. Exercise was associated with a significant reduction in visceral (*p* < 0.001), subcutaneous (*p* < 0.001) and intrahepatic fat (*p* < 0.001), as well as gamma-glutamyl transferase (GGT) levels (*p* < 0.001) in pediatric obesity [69]. Supervised exercise significantly reduced hepatic fat content compared with the control groups in younger individuals [70]. Exercise training for about 12 weeks induced an absolute reduction in intrahepatic TG levels of 3.31% (95%CI, −4.41 to −2.2) [71]. Exercise reduces intrahepatic TG levels independent of significant weight change (−2.16%; 95%CI, −2.87 to −1.44) but the benefits are substantially greater when weight loss occurs (−4.87%; 95%CI, −6.64 to −3.11). Furthermore, meta-regression identified a positive association between percentage weight loss and the absolute reduction in intrahepatic TG levels (β, 0.99; 95%CI, 0.62 to 1.36; *p* < 0.001). Furthermore, exercise training also improves hepatic insulin sensitivity.

#### 4.1.3. Diet and Exercise

A meta-analysis including RCTs assessed the effect of lifestyle-induced weight loss in MASLD. A ≥5% weight loss improved hepatic steatosis and a ≥7% weight loss also improved NAFLD activity score; however, fibrosis was unchanged [72]. Interventions combining exercise and diet led to a decrease in ALT levels (*p* < 0.01) and an improvement in NAFLD activity score [68]. In a systematic review and meta-analysis that assessed the effect of lifestyle changes on metabolic parameters in patients with MASLD, compared with conventional treatment, combined exercise with diet seemed to elicit greater reductions in ALT levels (mean difference (MD), −13.27; 95%CI, −21.39 to −5.16), AST levels (MD, −7.02; 95%CI, −11.26 to −2.78) and homeostatic model assessment insulin resistance (HOMA-IR) (MD, −2.07; 95%CI, −2.61 to −1.46) than diet (ALT MD, −4.48; 95%CI, −1.01 to −0.21; HOMA-IR MD, −0.61; 95%CI, −1.01 to −0.21) and exercise (ALT and AST non-significant; HOMA-IR MD, −0.46; 95% CI, −0.8 to −0.12) alone [73].

In conclusion, combining exercise and diet interventions may improve MASLD by greater degree than using diet or exercise alone.

### 4.2. Surgical Interventions

#### 4.2.1. Bariatric Surgery

Bariatric surgery has an important role in managing obesity. It can achieve significant weight loss, normalization of glucose tolerance [74] and reduce the cardiovascular risk and long-term mortality [75,76]. Bariatric surgery is associated with a significant reduction in the weighted incidence of a number of histological features of MASLD, including steatosis, fibrosis, hepatocyte ballooning and lobular inflammation [77].

#### 4.2.2. Intragastric Balloons

Intragastric balloons are safe and effective in inducing weight loss in obese patients. To review and analyze the available data of the effect of intragastric balloons on markers of MASLD and liver enzymes, a meta-analysis was performed [78]. ALT levels decreased by −10.02 U/L (95%CI, −13.2 to −6.8), GGT levels decreased by −9.82 U/L (95%CI, −12.9 to −6.8), and BMI decreased by −4.98 kg/m^2^ (95%CI, −5.6 to −4.4) with intragastric balloon therapy [79]. Hepatic steatosis evaluated by magnetic resonance imaging (MRI) was improved from baseline after 6 months of balloon therapy. The histological NAFLD activity score was lower 6 months after intragastric balloon treatment versus controls with sham endoscopy and diet (*p* = 0.03). In another meta-analysis, an improvement in steatosis was observed in 79.2% of patients and NAFLD activity score and HOMA-IR were improved in 83.5% and 64.5% of MASLD patients, respectively [79]. A reduction in liver volume, as measured by computed tomography scan, was observed in 93.9% of patients undergoing intragastric balloon placement.

## 5. Pharmacological Interventions for MASLD

Considering that the development of CV events determines the prognosis of MASLD patients [44], the ideal therapeutic agents for MASLD should reduce body weight, improve coronary risk factors and, if possible, reduce CV events in addition to improving liver function. Several anti-MASH candidate drugs have been developed that enable treatment via the modulation of distinct signaling cascades, including a series of drugs targeting PPAR subtypes (PPARα/δ/γ) that are considered to be attractive because they can regulate both systemic lipid metabolism and inflammation [80]. Recent data suggest that non-targeted treatment with fibrates (PPARa agonists) modestly reduces the risk of incident CV events. However, the effect of fibrate treatment may be particularly beneficial in patients with guideline-endorsed indications for therapy due to evidence of atherogenic dyslipidemia. To investigate the influence of fibrates on vascular risk reduction in persons with atherogenic dyslipidemia, a meta-analysis including six RCTs was performed. This meta-analysis showed that compared with placebo, the greatest benefit with fibrate treatment was seen in 7389 subjects with high TG levels, where fibrate therapy reduced the risk of vascular events by 25%, and in 5068 subjects with both high TG and low HDL-C levels, where the risk was reduced by 29% [81]. Here, we consider the effect of the selective PPARα modulator pemafibrate, which we recently reported to improve liver function in MASLD [82].

Sodium glucose cotransporter 2 inhibitors (SGLT2is) and glucagon-like peptide-1 receptor agonists (GLP-1RAs) have been shown to improve coronary risk factors, including body weight, and suppress the occurrence of CV events [83,84]. Here, we also consider the effects of such drugs in MASLD.

### 5.1. Pemafibrate

#### 5.1.1. Effects of Pemafibrate on Liver Enzymes, Hepatic Steatosis and Fibrosis

Pemafibrate is a novel member of the selective PPARα modulator family that was designed to have a higher PPARα agonistic activity and selectivity than existing PPARα agonists such as fibrates [85]. We previously reported that pemafibrate significantly reduced serum levels of AST, ALT and GGT and significantly increased serum albumin levels at 3, 6 and 12 months after the start of pemafibrate treatment in patients with hypertriglyceridemia, which was associated with a reduction in atherogenic dyslipidemia [82].

Recently, we reported that pemafibrate significantly reduced the hepatic steatosis index at 12 months after the start of pemafibrate [86]. The marker for hepatic fibrosis was significantly reduced by pemafibrate after 12 months [86]. The fibrosis 4 (FIB-4) index significantly decreased in patients with a baseline FIB-4 index ≥1.45 at 12 months after the start of pemafibrate [86]. To our knowledge, this is the first study to report that pemafibrate improved both the hepatic steatosis and fibrosis indexes. Pemafibrate was reported to improve liver fibrosis as assessed by MR elastography or FibroScan aspartate aminotransferase score [87,88].

#### 5.1.2. The Underlying Mechanisms for the Improvement of MASLD Using Pemafibrate

**Figure 2:**
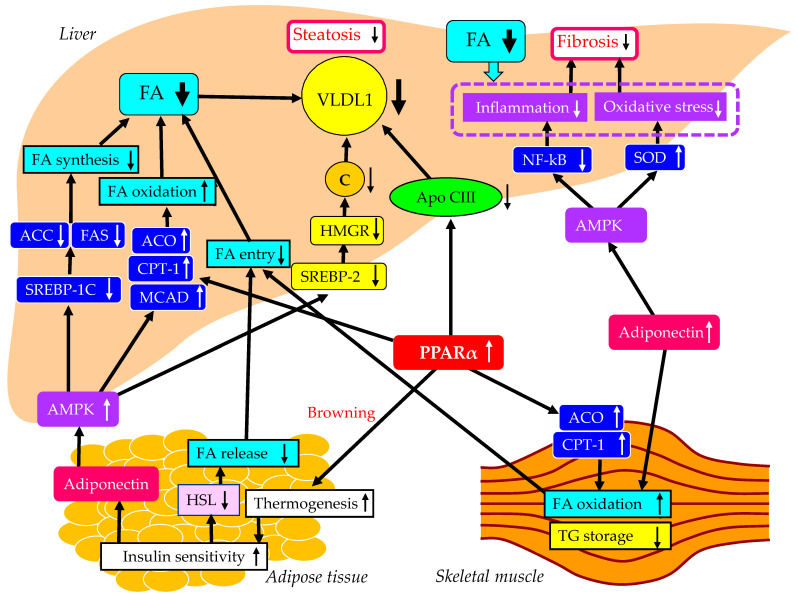
The underlying mechanisms for the improvement of MASLD using pemafibrate. Black arrows and white arrows pointing upward or downward indicate increases or decreases in expression and activity, respectively. Black solid lines indicate the effects of each metabolic event.

The underlying mechanisms for the improvement of MASLD using pemafibrate are shown in Figure 2. The altered properties of white adipose tissue due to obesity are associated with insulin resistance [89,90]. Brown fat increases energy expenditure by increasing thermogenesis and can utilize blood glucose and lipids, resulting in improved glucose and lipid metabolism [91], which leads to a reduction of FFA release from the adipose tissue. PPARα agonists can induce the browning of white adipose tissue [92], leading to an improvement in systemic insulin resistance. PPARα agonists enhance adiponectin production [93], which may also be beneficially associated with systemic insulin resistance [94]. The PPARα activation markedly stimulated the muscle and liver expression of two key enzymes involved in FA oxidation, CPT-1 and acyl-CoA oxidase (ACO) [95]. Moreover, the liver and muscle TG contents were significantly reduced by PPARα treatment [95].

Elevated FA oxidation in the skeletal muscle and the reduced FA release from the adipose tissue by PPARα agonists decrease FA entry into the liver and may result in a reduction in hepatic VLDL production. PPARα agonists reduce hepatic TG synthesis by decreasing apo CIII production [96]. Furthermore, treatment with PPARα agonists simulated the expression of ACO and CPT-1, leading to an increase in FA oxidation and a decrease in hepatic TG storage [97].

PPARα agonists enhance adiponectin production; adiponectin activates adenosine 5’-monophosphate (AMP)-activated protein kinase (AMPK) [94]. AMPK has long been regarded as a key regulator of energy metabolism, which is recognized as a critical target for MASLD treatment. AMPK activation reduces expression of the genes related to FA synthesis such as acetyl-CoA carboxylase (ACC) and FA synthase (FAS), by downregulating the mRNA of SREBP-1c [98]. AMPK activation increases the expression of genes related to FA oxidation, such as ACO, CPT-1 and medium-chain acyl-CoA dehydrogenase (MCAD) [98]. AMPK activation also inhibits the expression of SREBP-2 and its target genes, such as HMGR, which is the key enzyme in cholesterol biosynthesis [99]. Such increased FA oxidation and reduced FA and cholesterol production in the liver decrease hepatic VLDL production. Reduced hepatic VLDL accumulation may improve hepatic fibrosis by reducing inflammation and oxidative stress. Furthermore, AMPK activation improves inflammation by inhibiting NF-κB [100] and ameliorates oxidative stress by increasing the expression of superoxide dismutase (SOD) [101], which both contribute to a reduction of hepatic fibrosis.

#### 5.1.3. The Vasculoprotective Effects of Pemafibrate

PPARα agonists reduce hepatic TG synthesis by decreasing apo CIII production [96]. Furthermore, treatment with PPARα agonists simulated the expression of enzymes involved in FA oxidation, leading to a concomitant decrease in hepatic VLDL production [97]. PPARα agonists stimulate the activity of LPL, which further reduces VLDL levels [96]. As a result, there is an increase in HDL levels and a decrease in SdLDL and remnant lipoprotein levels [54]. PPARα agonists elevate HDL-C levels via transcriptional induction of apo AI and apo AII formation [96]. Pemafibrate is also effective at reducing atherogenic postprandial hyperlipidemia [102].

In addition, PPARα agonists promote HDL-mediated cholesterol efflux from macrophages via enhanced expression of ABCA1 [103]. PPARα agonists have multiple beneficial effects on vascular integrity, such as anti-inflammatory effects and inhibitory effects on vasoconstriction [54]. Furthermore, PPARα agonists inhibit smooth muscle cell proliferation, adhesion of monocytes to endothelial cells and OxLDL formation. PPARα agonists have a beneficial effect on the procoagulant state.

In a meta-analysis investigating the influence of fibrates on vascular risk reduction in subjects with atherogenic dyslipidemia [81], compared with placebo, the greatest benefit with fibrate treatment was observed in subjects with high TG levels; fibrate therapy reduced the risk of vascular events by 25%. Very recently, the PROMINENT trial was performed to study whether pemafibrate reduces the CV risk in patients with type 2 diabetes, mild-to-moderate hypertriglyceridemia and low HDL-C and LDL-C levels [104]. The median follow-up was 3.4 years. Pemafibrate reduced serum TG levels by 26.2%, VLDL-C levels by 25.8%, remnant cholesterol levels by 25.6% and apo CIII levels by 27.6% compared with placebo after 4 months. However, the incidence of CV events was not lower among those who received pemafibrate than among those who received placebo. Further studies should be performed to evaluate the effect of pemafibrate on CVD in the future.

### 5.2. SGLT2i

#### 5.2.1. Effects of SGLT2i on Liver Enzymes, Hepatic Steatosis and Fibrosis

SGLT2 mediates approximately 90% of the active renal glucose reabsorption in the proximal tubule of the kidney [105]. SGLT2is decrease plasma glucose without an increase in insulin secretion by reducing renal glucose reabsorption [106], which is favorable for body weight reduction and the improvement of coronary risk factors [107].

We previously reported that SGLT2is significantly reduced the serum levels of AST and ALT at 3 and 6 months after the start of the SGLT2i treatment in patients with type 2 diabetes [108,109]. Hepatic fibrosis can be evaluated by using the noninvasive FIB-4 index, which was reported as a useful index in MASLD [110]. A FIB-4 ≥2.67 score had an 80% positive predictive value for the identification of advanced hepatic fibrosis [110]. We found that the FIB-4 index was significantly decreased at 12 months after the start of SGLT2i treatment in a high-risk (FIB-4 ≥ 2.67) group for advanced hepatic fibrosis [111]. The correlations between the change in the FIB-4 index during the 12-month SGLT2i treatment was correlated inversely with the baseline FIB-4 index. We also retrospectively studied 568 patients with MASLD and type 2 diabetes. At 96 weeks, the mean FIB-4 index had significantly decreased (from 1.79 ± 1.10 to 1.56 ± 0.75) in the SGLT2i group but not in the pioglitazone group [112]. Another marker for hepatic fibrosis, the aspartate aminotransferase-to-platelet ratio index (APRI), significantly decreased in both groups. The body weight of the SGLT2i-treated group decreased by 3.2 kg; however, that of the pioglitazone group increased by 1.7 kg.

To summarize the evidence on the effects of SGLT2i treatment on liver structure and function, a meta-analysis of 20 RCTs was performed that showed that SGLT2is induced a significant decrease in serum ALT (−7.43 U/L, 95%CI; −12.14 to −2.71; *p* < 0.01), AST (−2.83 U/L; 95%CI, −4.71 to −0.95; *p* < 0.01) and GGT levels (−8.21 U/L; 95%CI, −9.52 to −6.91, *p* < 0.01) compared with placebo or other oral antidiabetic drugs [113]. The other oral antidiabetic drugs included metformin, sulfonylurea or dipeptidyl peptidase-4 inhibitors. SGLT2i treatment was associated with a decrease in liver steatosis (−3.39%; 95%CI, −6.01 to −0.77; *p* < 0.0.1) [113]. Improvements in such liver enzymes and liver fat content were also observed in other meta-analyses [114,115,116,117].

Type IV collagen is one of the extracellular matrices that are produced by hepatic fibroblasts. The 7S domain in the N-terminus of type IV collagen is inserted into tissues and released into the blood by turnover in connective tissues. Therefore, the serum 7S domain level increases in parallel with the amount of fibrosis and in synthesis from stellate cells and myofibroblasts following increased liver fibrosis [118]. In Japan, type IV collagen 7S is now widely used for assessing the extent of hepatic fibrosis. Elevated serum ferritin has been the main manifestation of disturbed iron homeostasis in chronic liver diseases and was reported to be independently associated with advanced liver fibrosis in patients with MASLD [119,120,121]. To analyze the effects of SGLT2i treatment on the indexes of liver fibrosis in patients with type 2 diabetes complicated with MASLD, and also to observe the effects on liver enzymes and liver fat, a meta-analysis was performed. This meta-analysis showed that SGLT2i treatment significantly reduced the levels of FIB-4 (MD, 0.25; 95%CI, −0.39 to −0.11; *p* = 0.0007), serum type IV collagen 7s (MD, 0.32; 95%CI −0.59 to −0.04; *p* = 0.02) and ferritin (MD, 26.7; 95%CI, 50.64 to 2.76, *p* = 0.03) [122].

In recent years, the use of transient elastography with Fibroscan^®^ [Echosens, Paris, France] equipment to obtain controlled attenuation parameters and liver stiffness measurements has been seen as a promising tool for the noninvasive quantification of hepatic steatosis and fibrosis, respectively [123,124], and has shown low failure (3.2%), high reliability (>95%) and high reproducibility [125]. In a meta-analysis, when compared with a control group, SGLT2i treatment significantly reduced liver stiffness (MD, −0.50; 95%CI, −0.99 to −0.01; *p* = 0.002), controlled attenuation parameters (MD, −0.74; 95%CI, −1.21 to −0.27; *p* = 0.005), serum ferritin levels (MD, −1.36; 95% CI [−2.14, −0.57], *p* = 0.0008), serum type IV collagen 7S levels (MD, −0.66; 95%CI, −1.2 to −0.12; *p* = 0.0004) and the FIB-4 index (MD, −0.37; 95%CI, −0. 74 to −0.01; *p* = 0.03) [126].

#### 5.2.2. The Underlying Mechanisms for the Improvement of MASLD Using SGLT2is

**Figure 3:**
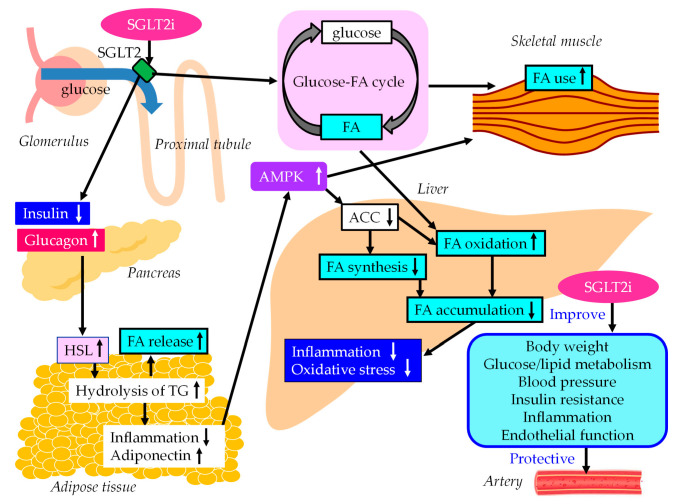
The underlying mechanisms for the improvement of MASLD and vascular protection using SGLT2is. Black arrows and white arrows pointing upward or downward indicate increases or decreases in expression and activity, respectively. Black solid lines indicate the effects of each metabolic event.

The underlying mechanisms for the improvement of MASLD and vascular protection using SGLT2is are shown in Figure 3. SGLT2i treatment decreases plasma glucose without an increase in insulin secretion by reducing renal glucose reabsorption, resulting in an increase in the ratio of glucagon to insulin, which activates HSL in adipose tissue [127]. As a result, FA release from adipose tissue increases due to an increase in the hydrolysis of the stored TG, which reduces fat mass with a diminished adipocyte size, resulting in an improvement in insulin resistance. An increase in the hydrolysis of TG increases serum FFA levels; however, such increased FA levels may be promptly used by skeletal muscles and the liver.

Inflammatory biomarkers may play vital roles in the pathophysiology of diabetes and diabetic cardiorenal complications. SGLT2is have potential cardiovascular- and renal-protective effects in type 2 diabetes. The aim of this meta-analysis was to quantify the effects of SGLT2is on biomarkers of inflammation in RCTs. The meta-analysis showed that SGLT2i treatments reduce C-reactive protein (CRP) levels (MD, 0.25; 95%CI, −0.47 to −0.03, *p* = 0.02) and improve adiponectin levels (MD, 0.28; 95%CI, 0.15 to 0.41, *p* < 0.001) compared with placebo [128]. An increase in adiponectin levels has beneficial effects on glucose and lipid metabolism via activation of AMPK [94]. The activation of AMPK is also associated with the improvement of MASLD via SGLT2i.

SGLT2i treatment shifted the energy metabolism towards FA utilization and elevated AMPK and ACC phosphorylation in skeletal muscle in diet-induced obese mice [129]. Furthermore, SGLT2i treatment induced a negative energy balance by excreting glucose into the urine, which may induce alteration of the glucose–FA cycle [130]. The fundamental concept of the glucose–FA cycle is the reciprocal substrate competition between glucose and FA in oxidative tissues such as skeletal muscles. SGLT2i-mediated alteration of the glucose–FA cycle may increase FA metabolism in the skeletal muscle. SGLT2i treatment reduces FA accumulation in liver, which reduces inflammation and oxidative stress and results in an improvement of MASLD.

SGLT2is differ from GLP-1RAs and pemafibrate in their effects on HSL in the adipose tissue: SGLT2is increase HSL activity, whereas GLP-1RAs and pemafibrate decrease HSL activity in the adipose tissue. SGLT2i treatment can still potentially lower steatosis and FA accumulation in the liver when there is a higher flux of FA from adipose tissue to the liver. The deficiency of carbohydrates caused by SGLT2i treatment decreases the circulating insulin levels. This promotes lipolysis, and the breakdown of fat becomes the major source of energy. The hepatic energy metabolism is regulated so that, under these circumstances, ketone bodies are generated from the β-oxidation of FAs and are secreted as ancillary fuel, in addition to gluconeogenesis [131]. SGLT2i treatment increases the β-oxidation of FAs, which creates a situation where FAs do not accumulate even if the influx of FAs into the liver increases.

#### 5.2.3. The Vasculoprotective Effects of SGLT2i

To assess the efficacy and safety of SGLT2i treatment in adults with type 2 diabetes, a meta-analysis was performed, which showed that SGLT2i treatment reduced hemoglobin A1c (MD, −0.66%; 95% CI, −0.73% to −0.58%), body weight (MD, −1.80 kg; 95%CI, −3.50 to −0.11 kg) and systolic blood pressure (MD, −4.45 mmHg; 95%CI, −5.73 to −3.18 mmHg) [132]. Meta-analyses also showed a significant increase in HDL-C levels and a significant decrease in TG levels [133,134]. Very recently, we reported that reduced levels of fasting apo B48, remnant lipoprotein cholesterol and non-HDL-C caused by SGLT2i treatment suggest the possible beneficial effect of SGLT2is in atherogenic postprandial hyperlipidemia [102].

SGLT2is have been shown to improve endothelial dysfunction, as assessed by flow-mediated vasodilation, in individuals at high risk of CVD [135]. SGLT2is have been shown to improve oxidative stress, inflammation, mitochondrial dysfunction, glucotoxicity (such as the advanced signaling of glycation end products) and nitric oxide bioavailability. Very recently, a subanalysis of a meta-analysis showed that SGLT2i treatment significantly reduced atherosclerotic major adverse cardiovascular events (MACEs) in subjects with both chronic kidney disease and type 2 diabetes without established ASCVD [136].

### 5.3. GLP-1RAs

#### 5.3.1. Effects of GLP-1RAs on Liver Enzymes, Hepatic Steatosis and Fibrosis

Recently, we reported that 12-month dulaglutide therapy significantly improved serum GGT levels and NAFLD activity score in patients with type 2 diabetes [137]. Meta-analyses showed that GLP-1RA improved liver enzymes [138] and liver histology scores for steatosis and fibrosis [139] and liver fat content using MRI-based techniques [140].

#### 5.3.2. The Underlying Mechanisms for MASLD Treatment using GLP-1RAs

**Figure 4:**
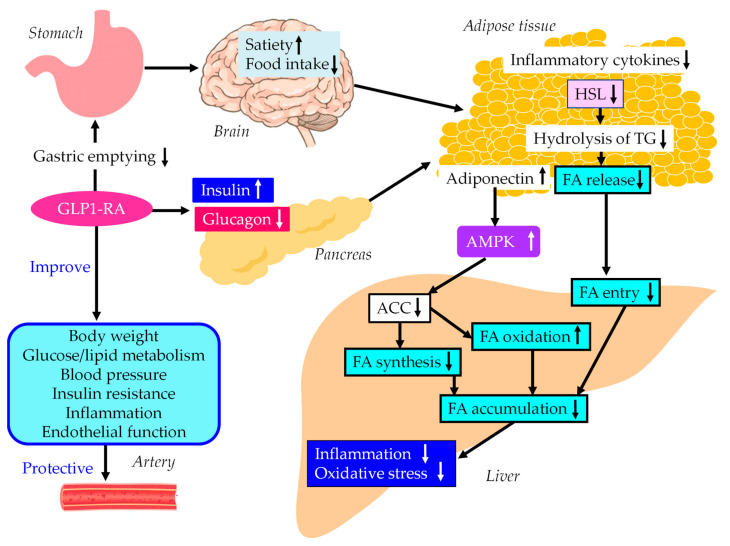
The underlying mechanisms for MASLD treatment and vascular protection using GLP-1RAs. Black arrows and white arrows pointing upward or downward indicate increases or decreases in expression and activity, respectively. Black solid lines indicate the effects of each metabolic event.

The underlying mechanisms for MASLD treatment and vascular protection using GLP-1RA are shown in Figure 4. GLP-1RAs increase pancreatic insulin secretion and decreases glucagon in a glucose-dependent manner and delays gastric emptying, which suppresses postprandial hyperglycemia and appetite, resulting in reductions in energy intake and body weight [141,142,143]. Intestinal GLP-1 is an endogenous satiation signal; its eating effects are primarily mediated by vagal afferents [144]. Increases in insulin secretion and decreases in glucagon secretion reduce HSL activity, resulting in decreases in the hydrolysis of TG and FA release from adipose tissue, which reduces FA entry to the liver.

A meta-analysis and systematic review were conducted to examine the effects of GLP-1RAs on the clinical biomarkers of inflammation and oxidative stress in patients with type 2 diabetes. The meta-analysis showed that GLP-1RA treatment led to significant reductions in CRP and TNF-α levels and a significant increase in adiponectin levels compared with standard diabetes therapies or placebo [145]. GLP-1RAs increase adiponectin levels, which activates AMPK. AMPK activation may also contribute to an improvement in MASLD symptoms by using GLP-1RA.

#### 5.3.3. The Vasculoprotective Effects of GLP-1RAs

GLP-1RA treatment achieved a greater systolic blood pressure reduction than comparator therapy (MD, 2.22 mmHg; 95%CI, −2.97 to −1.47). In the pooled analysis, GLP-1RAs had beneficial effects on weight loss (MD, −2.56 kg; 95%CI, −3.12 to −2.00) and hemoglobin A1c reduction (MD, −0.41%; 95%CI, −0.78 to −0.04) [146]. Compared with the patients before the treatment, patients after the GLP-1RA treatment showed significantly reduced values for hemoglobin A1c, BMI, LDL-C and TG [147]. Furthermore, GLP-1RAs improved atherogenic postprandial hyperlipidemia [102]. In addition, GLP-1RAs have multiple vascular biological anti-atherogenic properties, such as improvement of endothelial function [84]. Since 2005, the cardioprotective effects of GLP-1 RAs have garnered attention. The cardioprotective effect could be an added benefit of the use of GLP-1 RAs. A systematic review and meta-analysis aimed at summarizing the observational studies that recruited individuals with type 2 diabetes that had had fewer CV events before enrolling in the research. The meta-analysis showed that GLP-1 RA therapy was associated with a significantly lower risk of MACEs, extended MACEs, all-cause mortality and CV mortality [148].

### 5.4. Comparison of the Efficacy of SGLT2i/GLP-1RA/PPAR Agonists in MASLD

The relative efficacy of SGLT2is and GLP-1RA for MASLD therapy has not been sufficiently investigated. A systematic review and network meta-analysis of RCTs using five SGLT2is and four GLP-1RAs showed that semaglutide, liraglutide and dapagliflozin all have a certain effect on MASLD, based on high confidence evidence from indirect comparisons. Moreover, semaglutide appears to have a therapeutic advantage over the other included medicines [149]. In a meta-analysis including 25 RCTs comparing GLP-1RAs and SGLT2is with controls in adult MASLD patients with or without type 2 diabetes, compared with SGLT2is, GLP-1RAs significantly decreased visceral fat (−0.560, 95%CI, −0.961 to −0.131) and TG levels (−0.607, 95%CI, 1.095 to −0.117) [150]. A systematic review reported that PPAR agonists, such as pioglitazone and lanifibranor, and GLP-1RAs (mostly liraglutide and semaglutide) improved the individual histological features of MASH (steatosis, ballooning, lobular inflammation) or achieved resolution of MASH without a worsening of fibrosis. SGLT2is (mostly empagliflozin and dapagliflozin) reduced liver fat content, as assessed via MRI-based techniques [151]. There are no such data using pemafibrate. Head-to-head studies are needed to provide more confidence in clinical decision making.

### 5.5. Effects of Combination Therapy Using SGLT2is and GLP-1RAs in MASLD

To our knowledge, only our study has investigated the effects of combination therapy using SGLT2is and GLP-1RAs. We found that 12-month dulaglutide therapy significantly improved serum GGT levels and NAFLD activity score in patients with type 2 diabetes [137]. Although a significant improvement in GGT levels was not observed in patients treated with GLP-1RAs without SGLT2is (n = 69), a significant improvement in GGT levels was obtained in patients treated with a GLP-1RA and an SGLT2 (n = 52). Furthermore, the FIB-4 index tended to decrease from 1.74 ± 1.33 to 1.62 ± 1.10 (*p* = 0.088) in patients treated with GLP-1RAs and SGLT2is, whereas patients without SGLT2i treatment showed a non-significant increase in FIB-4 index, from 1.71 ± 1.02 to 1.75 ± 1.12 (*p* = 0.547). The combination therapy of SGLT2is and GLP-1RAs may be a promising therapeutic option for MASLD.

### 5.6. Effects of the Combination Therapy of SGLT2is and Pemafibrate on MASLD

To our knowledge, there are only two studies that have investigated the effects of the combination therapy of SGLT2is and pemafibrate including our study. The other group’s study was a pilot study with only seven patients. In their study, MASLD patients complicated with type 2 diabetes treated with pemafibrate for >1 year were included, in whom prior treatment with an SGLT2i >1 year failed to normalize serum ALT levels [152]. During the one year before starting pemafibrate therapy, the therapy did not significantly change hepatic enzymes. All patients received pemafibrate 0.1 mg twice daily. During one year of pemafibrate therapy, serum levels of TG, AST, ALT, GGT and Mac-2 binding protein glycosylation isomer, which is the marker for liver fibrosis, were significantly improved (*p* < 0.05).

We reported that pemafibrate significantly reduced the serum levels of AST, ALT and GGT and significantly increased serum albumin levels at 3, 6 and 12 months after the start of pemafibrate treatment in patients with hypertriglyceridemia (n = 246), with an improvement in atherogenic dyslipidemia [82]. We investigated the effects of a combination of pemafibrate and an SGLT2i by dividing patients into those treated with pemafibrate and an SGLT2i (n = 63) and those treated with pemafibrate without an SGLT2i (n = 183). There were no significant differences in changes in ALT, GGT and albumin between the two groups. Although a significant improvement in AST was not observed in patients treated without an SGLT2i at any time, a significant improvement in AST was observed in patients treated with pemafibrate and an SGLT2i at 3 and 12 months after the start of pemafibrate. The reversal of the AST/ALT ratio to >1 has been consistently reported to predict the presence of more advanced liver fibrosis [153]. The marker for liver fibrosis, APRI, was calculated with the formula: AST/upper limit of normal range of AST/platelet count × 100 [154]. Such a favorable effect of the combination of pemafibrate and an SGLT2i on the change in AST may show that this combination therapy can be a promising therapeutic option for MASLD.

## 6. The Limitations of Our Review

The section for surgical interventions and pharmacological interventions is incomplete and includes only a few selected surgical approaches for obesity and only a few of the approved medications for either obesity or type 2 diabetes. This must be clearly stated instead of providing a very narrow point of view that only focuses on the authors’ previous studies, including SGLT2i alone or in combination with other approved medications for type 2 diabetes.

## 7. Conclusions

**Figure 5:**
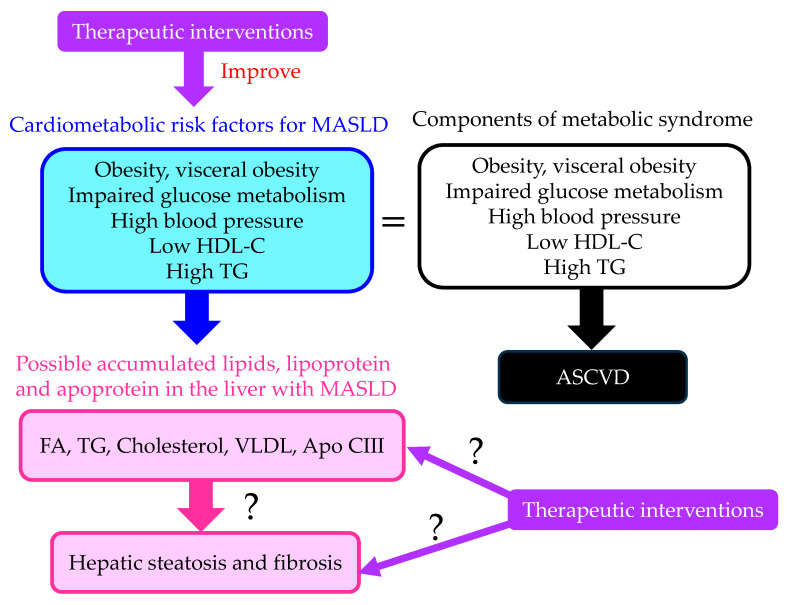
The summary of this review.

The summary of our review is shown in Figure 5. MASLD is a high-risk condition for both liver fibrosis and ASCVD. Therefore, therapeutic strategies to prevent both liver fibrosis and ASCVD are required for the treatment of MASLD. Therapeutic interventions that improve cardiometabolic risk factors may be beneficial for an improvement in MASLD. The effects of such therapeutic interventions on lipid, lipoprotein and apoprotein accumulation in the liver and on hepatic steatosis and fibrosis still remain unelucidated. Which lipid factor is crucial for developing MASLD also remains largely unknown. These issues should be studied in the future.

## Data Availability

Not applicable.

## Abbreviations

ACC	acetyl-CoA carboxylase
ACO	acyl-CoA oxidase
ALT	alanine aminotransferase
APRI	aminotransferase-to-platelet ratio index
AST	aspartate aminotransferase
ASCVD	atherosclerotic cardiovascular disease
AMPK	adenosine monophosphate-activated protein kinase
BMI	body mass index
C	cholesterol
CETP	cholesterol ester transfer protein
CI	confidence interval
CM	chylomicron
CMR	chylomicron remnant
CPT-1	carnitine palmitoyl-transferase 1
CRP	C-reactive protein
CVD	cardiovascular diseases
GGT	gamma-glutamyl transferase
FA	fatty acid
FAS	fatty acid synthase
FFA	free fatty acid
FIB-4	fibrosis-4
GLP-1RA	glucagon-like peptide-1 receptor agonists
HDL-C	high-density lipoprotein cholesterol
HMGR	3-hydroxy-3-methyl-glutaryl-CoA reductase
HL	hepatic lipase
HOMA-IR	homeostasis model assessment of insulin resistance
HR	hazard ratio
HSL	hormone-sensitive lipase
IDL	intermediate-density lipoprotein
IL	interleukin
IRS-2	insulin receptor substrate 2
LDL	low-density lipoprotein
LPL	lipoprotein lipase
LRP1	LDL receptor-related protein 1
MACE	major adverse cardiovascular events
MASH	metabolic-dysfunction-associated steatohepatitis
MASLD	metabolic-dysfunction-associated steatotic liver disease
MCAD	medium-chain acyl-CoA dehydrogenase
MD	mean difference
MRI	magnetic resonance imaging
MTP	microsomal TG transfer protein
NAFLD	nonalcoholic fatty liver disease
NASH	nonalcoholic steatohepatitis
NF-κB	nuclear factor-κB
NPC1L1	Niemann-Pick C1-like 1
OR	odds ratio
OxLDL	oxidized LDL
PPAR	peroxisome proliferator-activated receptor
RCTs	randomized controlled trials
Rem	remnant lipoproteins
ROS	reactive oxygen species
SdLDL	small dense LDL
SGLT2i	sodium-glucose cotransporter 2 inhibitors
SOD	superoxide dismutase
SR-BI	scavenger receptor class B type I
SREBP	sterol regulatory element binding protein
TNF-α	tumor necrosis factor-alpha
TG	triglyceride
VLDL	very low-density lipoprotein
WC	waist circumference
